# Recent Progress in the Development of Indole-Based Compounds Active against Malaria, Trypanosomiasis and Leishmaniasis

**DOI:** 10.3390/molecules27010319

**Published:** 2022-01-05

**Authors:** Paulo A. F. Pacheco, Maria M. M. Santos

**Affiliations:** Research Institute for Medicines (iMed.ULisboa), Faculty of Pharmacy, Universidade de Lisboa, 1649-003 Lisbon, Portugal; ppacheco@farm-id.pt

**Keywords:** synthetic indole, inhibitors, neglected diseases, malaria, trypanosomiasis, leishmaniasis

## Abstract

Human protozoan diseases represent a serious health problem worldwide, affecting mainly people in social and economic vulnerability. These diseases have attracted little investment in drug discovery, which is reflected in the limited available therapeutic arsenal. Authorized drugs present problems such as low efficacy in some stages of the disease or toxicity, which result in undesirable side effects and treatment abandonment. Moreover, the emergence of drug-resistant parasite strains makes necessary an even greater effort to develop safe and effective antiparasitic agents. Among the chemotypes investigated for parasitic diseases, the indole nucleus has emerged as a privileged molecular scaffold for the generation of new drug candidates. In this review, the authors provide an overview of the indole-based compounds developed against important parasitic diseases, namely malaria, trypanosomiasis and leishmaniasis, by focusing on the design, optimization and synthesis of the most relevant synthetic indole scaffolds recently reported.

## 1. Introduction

Although parasitic diseases, such as malaria, trypanosomiasis and leishmaniasis, have been neglected for a long time, research interest and investments have intensified in recent years [1]. In fact, the elimination of these diseases is proposed as a priority in the third goal of the United Nations 2030 Agenda for Sustainable Development [2,3]. However, there is still a long way to go. Together, neglected diseases (NTDs) and malaria caused 747,000 deaths worldwide in 2019 and represented an estimated burden of about 62.9 million disability-adjusted life years (DALYs) [4]. Despite advances in control and prevention, it is estimated that these diseases still affect a large contingent of people in social and economic vulnerability, especially in tropical and subtropical regions. Moreover, human migration and climate changes have impacted the distribution and transmission pattern of some of these diseases, making them a public health problem also for more developed regions [5,6].

Among NTDs, trypanosomiasis and leishmaniosis are vector-borne diseases caused by protozoa of the class Kinetoplastea and family Trypanosomatidae, which includes different monoflagellated parasites [7]. The genus *Trypanosoma* is divided into two sections, depending on the mode of transmission: Salivaria (transmitted by saliva) and Stercoraria (transmitted by feces) [8]. Two examples of trypanosomes of medical importance are *Trypanosoma cruzi* (Stercoraria), the causative agent of Chagas disease (CD), and *Trypanosoma brucei* (Salivarian), which causes sleeping sickness. Both diseases are responsible for high mortality, morbidity and economic burden in sub-Saharan Africa and Latin America [9,10].

**Figure 1:**
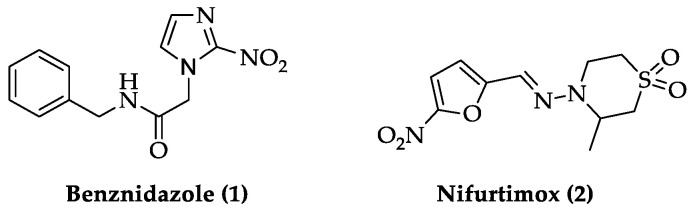
Currently available drugs in clinical use for American trypanosomiasis management.

Chagas disease, also known as American trypanosomiasis (AT), is a life-threatening zoonosis caused by the protozoan *T. cruzi* and that is endemic in 21 countries. It is transmitted to humans mainly by the bite of triatomine bugs or by ingestion of contaminated food [11,12]. Transmission can also occur in nonendemic areas by nonvectorial routes such as blood transfusions, congenital infection and organ transplantation [12,13]. The latest World Health Organization (WHO) reports estimate that 6 to 7 million people worldwide are infected with *T. cruzi* and another 75 million are at risk of infection due to under-diagnosis and inappropriate healthcare [14]. Since AT can be associated with progressive disability and early mortality, often in the most productive population, it also represents a serious economic burden in regions that already struggle with profound socioeconomic issues. The estimated global economic burden reaches USD 7.19 billion per year [7,15]. Despite AT having been discovered 112 years ago, AT treatment remains quite limited [16]. To date, there are only two available drugs, benznidazole (**1**) and nifurtimox (**2**) (Figure 1), approved for clinical use more than 50 years ago [17]. Since then, little progress has been made in this area. Both drugs have variable effectiveness, depending on the clinical stage of the disease. They are nearly 100% effective in curing or reducing parasitic burden in acute or early phases, in congenital cases and in reactivation in immunosuppressed patients [18]. However, their effectiveness is very restricted in chronic CD, and their advantages of use in these patients are still debatable [17]. These medications are also contraindicated during pregnancy or for people with renal or liver failure [17]. Furthermore, these drugs offer other inconveniences for chronic AT management such as requirement of high dosage and prolonged period of treatment to achieve therapeutic efficacy, resulting in a wide range of side effects and consequent therapy discontinuation [19,20]. In the case of nifurtimox, the low safety profile, due to the high risk of heart failure and gastrointestinal and neurological problems in prolonged treatment regimen, turned it a second-line option for AT in many endemic countries [21]. Another problem associated with the lack of therapeutic options for chronic patients is the need for adjunctive therapy for the management of cardiac, digestive or neurological complications [11].

**Figure 2:**
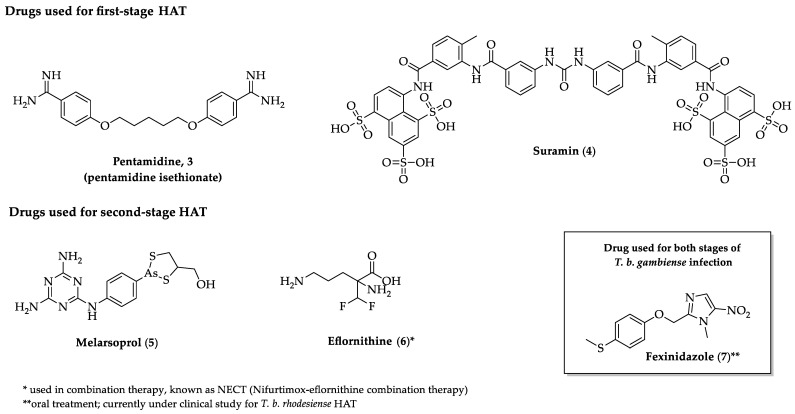
Approved drugs for human African trypanosomiasis (HAT) treatment.

Sleeping sickness, or human African trypanosomiasis (HAT), is another potentially disabling and fatal disease of medical and veterinary importance that is endemic in 36 sub-Saharan African countries [22,23]. This disease affects mainly rural populations in remote areas with very limited access to proper health assistance, which complicates the epidemiological surveillance scenario, diagnosis and treatment [24]. Although the sustained effort to control the disease has significantly impacted the disease incidence, the estimated population at risk accounts for 65 million people [24]. The main route of transmission is the bite of blood-sucking tsetse fly species (*Glossina* spp.) which makes HAT a focal disease [25]. The etiological agents are protozoa of two subspecies of *Trypanosoma brucei*, which cause different pathologies with specific epidemiology and geographical distribution: *Trypanosoma brucei gambiense* and *Trypanosoma brucei rhodesiense* [9]. *T. b. gambiense* has humans as main reservoir host and is transmitted by tsetse flies of the species *Glossina palpalis*. It is predominant in 24 countries in Western and Central Africa and causes a slow-progressing anthroponotic disease that can stay at an asymptomatic state for a long period [26]. This disease variant represents most of the reported HAT cases [24]. On the other hand, *T. b. rhodesiense* is transmitted to humans by tsetse flies of the *Glossina morsitans* group and is concentrated in 13 countries in Eastern and Southern Africa [24]. The main reservoir for *T. b. rhodesiense* is ungulates and cattle, with humans being occasional hosts, which makes this disease extremely difficult to eradicate [27]. For this reason, strategies for HAT eradication have focused on *T. b. gambiense* control [26,27]. The infection caused by *T. b. rhodesiense* manifests as a fast-progressing, acute and severe syndrome [28]. The clinical manifestation of HAT depends on different factors such as the parasite subspecies, host immune response or disease stage. In general, the disease progresses through two stages: a hemolymphatic stage, which is characterized by the proliferation and spread of trypanosomes within the blood and lymphatic system, followed by a meningoencephalitic stage, in which parasites cross the blood–brain barrier and invade the central nervous system [28]. Regardless of the parasite subspecies, if left untreated HAT can lead to coma and fatal outcome. As for American trypanosomiasis, HAT therapy relies on a limited set of drugs (Figure 2), most of them developed many years ago and presenting a certain degree of toxicity [29]. The most critical example is the melarsoprol (**5**), an organic arsenical drug used for treatment of second-stage *T. b. rhodesiense* disease, which can lead to encephalopathic syndrome in 5–18% of treated patients and to fatality in 3–10% [30]. Another complicating factor in the treatment of HAT is the route of administration of these drugs, mostly parenteral, which creates logistical difficulties in regions with scarce health infrastructure [28]. Therefore, a drug preferably oral, safe and effective for late-stage disease is highly necessary to achieve the goal of HAT eradication.

**Figure 3:**
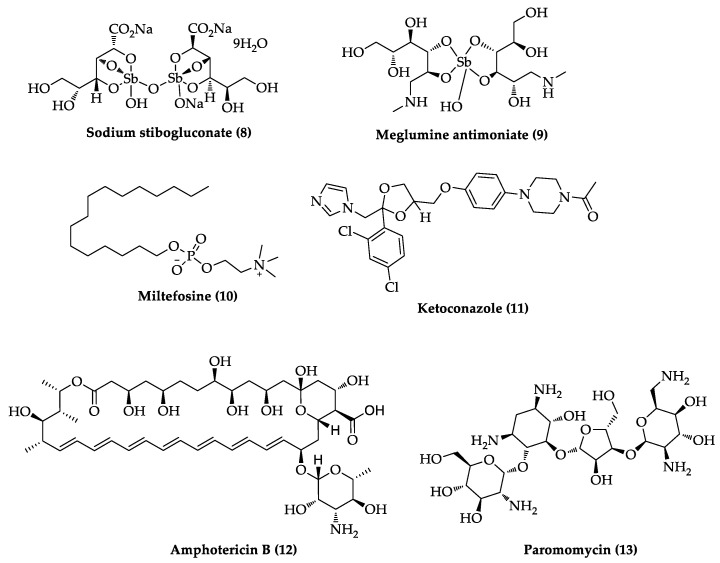
Currently available drugs for leishmaniasis treatment.

Other kinetoplastid diseases that have been long neglected in terms of chemotherapy development are the leishmaniases. They consist of a complex group of parasitic diseases that are caused by intracellular flagellate protozoa of the genus *Leishmania* and have multiple clinical manifestations [31]. To date, over 20 species of *Leishmania* parasites, from the subgenera *Leishmania* and *Viannia*, have been identified as infective for humans [32]. In general, they are divided into Old and New World parasites, according to their geographic dispersion [33]. The main mode of transmission is the bite of hematophagous phlebotomine sand flies (mainly of the genera *Phlebotomus* and *Lutzomyia*) [34]. Leishmaniases have virtually global distribution, being present in almost 100 countries and endemic in Africa, the Americas, Asia and the Mediterranean region [31]. An estimated 700,000 to 1 million new cases occur worldwide annually [35]. The strict association with poverty and its sociopolitical implications (e.g., malnutrition, poor housing or population displacement) has relegated these diseases to a considerable underdevelopment regarding new diagnostic methods and effective and safe chemotherapeutic agents [36]. Despite the diversity of causative agents, the clinical manifestations can be categorized into three main forms, cutaneous leishmaniasis, mucocutaneous leishmaniasis and visceral leishmaniasis (also known as kala-azar), that will differ in severity and geographical occurrence [37]. The cutaneous form is caused by the so-called Old World *Leishmania* species, such as *L. major*, *L. tropica* and *L. aethiopica*, or New World species such as *L. amazonensis*, *L. mexicana*, *L. braziliensis* and *L. guyanensis*. It is characterized by self-healing diffuse ulcerations on skin that can leave permanent scars, disability and social stigma [33,38]. Some patients infected with parasites from *Viannia* subgenus (i.e., *L. braziliensis* and *L. guyanensis*) can also evolve to the mucocutaneous form, which is characterized by disfiguring lesions in the face and destruction of mucous tissues in the mouth and nose [33]. The visceral leishmaniasis, caused by the Old World species *L. infantum* (*syn. L. chagasi*) and *L. donovani*, is the most lethal form, since it provokes serious dysfunctions in several organs, such as liver, spleen and bone marrow [33,39]. Unlike the other forms, visceral leishmaniasis presents high lethality in the absence of treatment [33,39]. In addition, coinfection with HIV even further complicates the management of visceral leishmaniasis [40]. Although the chemotherapy for leishmaniasis is more diverse than that for other kinetoplastid diseases, it suffers from the same limitations such as prolonged and high-cost treatment, low therapeutic index, leading to side effects, treatment discontinuation and reported emergence of drug resistance [20,31,33]. The first-line treatments for leishmaniasis (Figure 3) are pentavalent antimonials (e.g., sodium stibogluconate (**8**) and meglumine antimoniate (**9**)), but other alternatives such as pentamidine (**3**), miltefosine (**10**), ketoconazole (**11**), amphotericin B (**12**) and paromomycin (**13**) can be used [41]. It is important to highlight that none of these medications were designed specifically for leishmaniasis treatment and the need for new chemotherapeutic alternatives is high. 

In the absence of approved human vaccines to combat these groups of diverse kinetoplastid diseases, the control and eradication effort still relies largely on the use of small molecules targeting these parasites. Furthermore, vaccine development for protozoan diseases itself is a very challenging task due to the high diversity and complex nature of the parasites and host response [42,43]. An exception in this scenario is the recently approved RTS,S malaria vaccine targeting the deadly parasite *Plasmodium falciparum*, recommended for children in sub-Saharan Africa and in other regions of moderate or high malaria transmission [44]. Although it represents a breakthrough and paradigm shift in malaria control, some obstacles, such as a modest efficacy and complex dose regimen, still need to be addressed before the total success of reducing the burden of disease [45]. 

**Figure 4:**
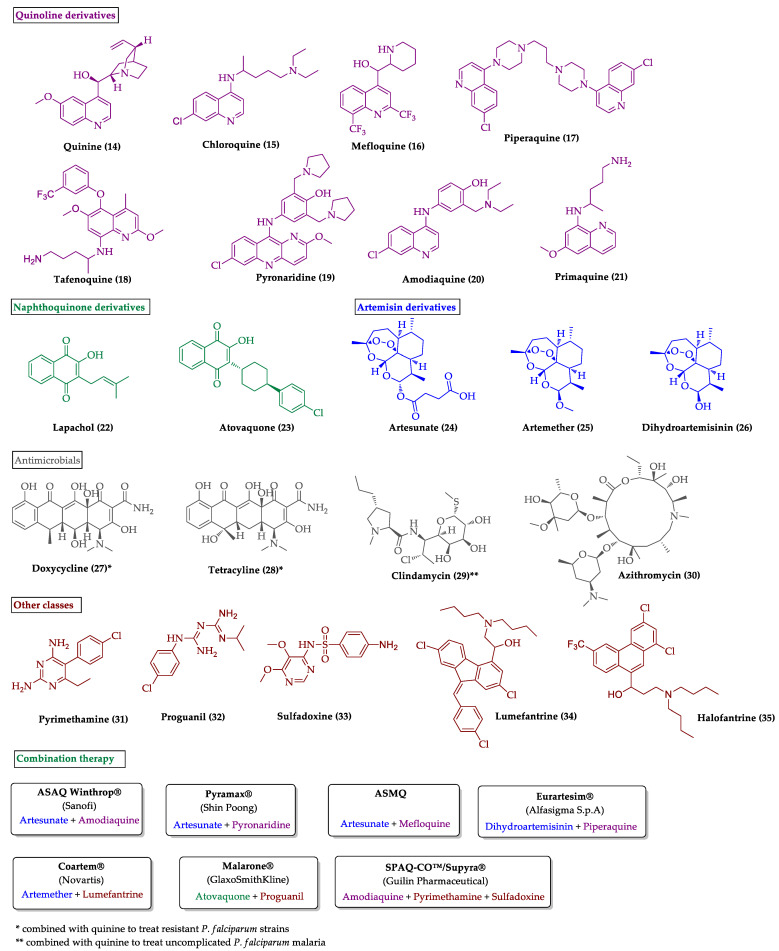
Antimalarial drugs and combination therapies in clinical use.

In this sense, the use of antimalarial drugs remains an essential tool to mitigate the burden of malaria. Despite some progress in control and prevention of this disease, malaria remains one of the most relevant parasitic diseases regarding morbidity and mortality worldwide [46]. It is estimated that half of the world population is at risk from Malaria. In 2019, there were an estimated 229 million cases of malaria in 87 endemic countries, with 94% of cases concentrated in the World Health Organization (WHO) African Region [46]. The malaria death rate (i.e., deaths/100,000 population at risk) has decreased between 2000 and 2019, but the rate of decline is experiencing a deceleration in recent years [46]. According to the latest WHO malaria report, the disease was responsible for 409,000 deaths globally (95% of them concentrated in only 31 countries) [46]. Malaria can be caused by six species of *Plasmodium* parasites [47]. Among them, *P. vivax* and *P. falciparum* are the most epidemiologically relevant species [48]. In the case of *P. falciparum* infection, lack of treatment can lead to severe illness and death [49]. In Figure 4, the available drugs for the treatment of malaria patients and the different options of combination therapies are presented. The existence of parasite strains of both species that are resistant to most conventional drug treatments is well established in several endemic areas [50,51,52,53]. Because of this, the best recommended treatment, particularly for uncomplicated *P. falciparum* malaria, involves the combination of a short-acting artemisinin derivative with one or more partner drugs with long-lasting effects and different mechanisms of action [54]. This regimen is known as artemisinin-based combination therapy (ACT) [54,55]. However, there have been reports of the emergence of *Plasmodium* strains resistant to artemisinin, which poses a great threat to all achievements in the combat against malaria in recent decades [56]. Therefore, it is urgently necessary to develop new antimalarial agents based on different chemical scaffolds and targeting multiple stages of parasite development to prevent the development of cross-resistance between similar chemical families. 

In this regard, the indole moiety has long proven to be one of the most promising chemotypes for the development of new and effective antiparasitic drugs [57]. In the following sections, we discuss the recent advances in the medicinal chemistry of indole-based compounds targeting these three relevant parasitic diseases.

## 2. Recent Development of Indole-Based Small Molecules Targeting Malaria, Trypanosomiasis and Leishmaniasis

### 2.1. Indole Derivatives with Antiplasmodial Activity

Several good reviews have been published in recent years that are focused on indole derivatives, mainly natural products, with antimalarial activity [58,59,60,61,62]. For this reason, in this section, we only focus on the most relevant indole synthetic derivatives reported in the last decade. 

**Figure 5:**
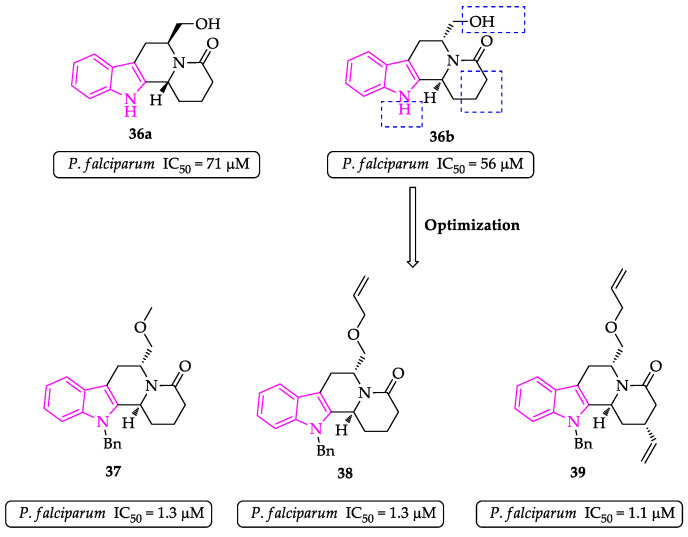
Optimization of the indoloisoquinoline scaffold.

In 2012, a SAR study for the indoloisoquinoline template, present in the natural product dihydrousambarensine, was reported. Several compounds derived from *L*- or *D*-tryptophan were prepared and evaluated against the Dd2 chloroquine-resistant *P. falciparum* strain. Optimization by chemical modification of the indole nitrogen, the hydroxymethyl substituent and the lactam ring led to three compounds with low micromolar activity (Figure 5). Results showed that (i) compounds derived from *D*-tryptophan were more active, (ii) aromatic groups were preferred on the indole nitrogen and (iii) introduction of allyl at the hydroxymethyl and at the piperidone ring led to an increase in activity [63].

**Scheme 1:**
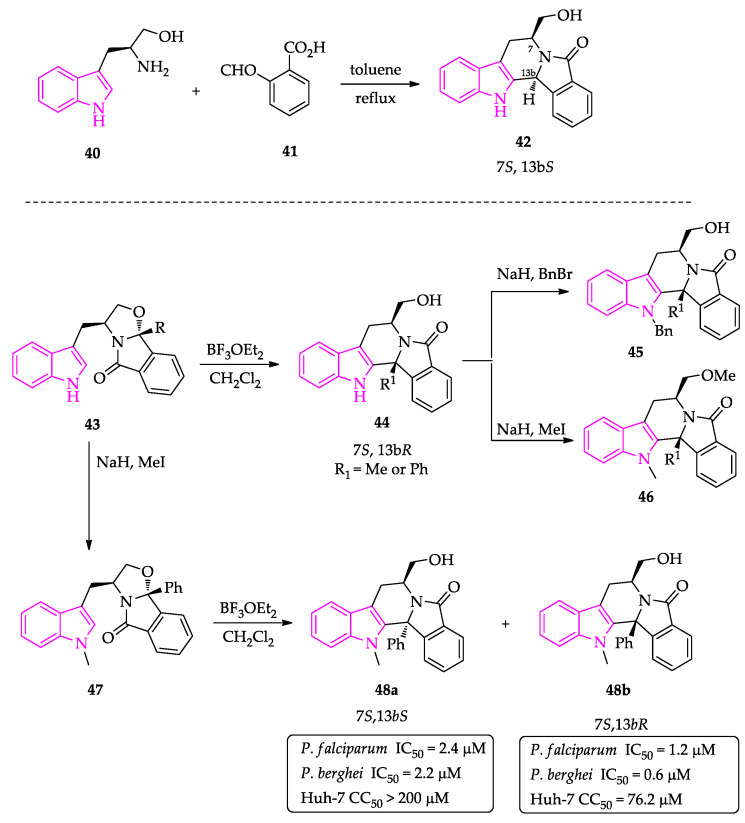
Synthesis of indolizinoindolones.

In 2015, a novel class of indole-based compounds (indolizinoindolones) with in vitro activity against blood- and liver-stage malaria parasites was developed. The rationale was to merge the isoindolinone and indole moieties in the same scaffold and evaluate the effect on antiplasmodial activity. The enantiopure target indolizinoindolones were synthesized by cyclocondensation reaction of (*S*)- or (*R*)-tryptophanol and 2-acyl benzoic acids, followed by Pictet–Spengler cyclization. Starting from (*S*)-tryptophanol, only the 7*S*,13b*R* stereoisomer was detectable by NMR spectroscopy. However, cyclocondensation of (*S*)-tryptophanol with 2-formylbenzoic acid led directly to 7*S*,13*bS* stereoisomer (Scheme 1). The effect on activity of hydroxymethyl *O*-protection and indole *N*-protection was also evaluated [64]. 

The synthesized indolizinoindolones showed activities in the low micromolar range against erythrocytic stages of the human malaria parasite *P. falciparum* (chloroquine-resistant W2 strain) and liver stages of the rodent parasite *Plasmodium berghei* [64]. Protection of the indole nitrogen atom with a benzyl group had a small impact on activity, and most compounds derived from (*S*)-tryptophanol were more active than the corresponding enantiomers derived from (*R*)-tryptophanol [64]. The substitution of the isoindolinone moiety with a pyrrolidone group resulted in a loss of activity, indicating that the isoindolinone probably is relevant for activity. The (*S*)-tryptophanol-derived isoindolinones **48a** and **48b** were the most active derivatives, combining excellent activity against both stages of *Plasmodium*’s life-cycle with IC_50_ values in the low micromolar range, having low cytotoxicity against human liver cell line and good metabolic and chemical stability (Scheme 1) [64].

**Scheme 2:**
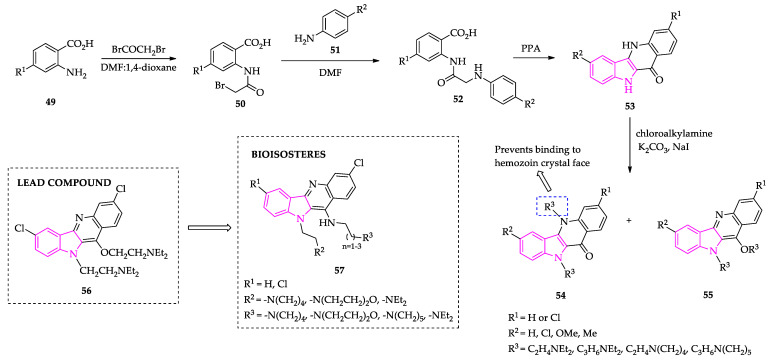
Synthesis of bis-alkylamine indolo[3,2-*b*]quinolines tested against *P. falciparum*.

The indolo[3,2-*b*]quinoline alkaloid cryptolepine is a natural product with antimalarial activity; it binds to heme and inhibits hemozoin formation inside the parasite digestive vacuole. To optimize the activity of this natural product, bis-alkylamine indolo[3,2-*b*]quinolines were synthesized and evaluated against chloroquine-resistant and sensitive strains of *P. falciparum*. The synthetic process to obtain the bisalkylamines (Scheme 2) started with the reaction between anthranilic acids and bromoacetyl bromide, followed by nucleophilic substitution using the appropriate aniline. Bicyclization, promoted by polyphosphoric acid (PPA), afforded the corresponding quindolones, which were then reacted with different chloroalkylamines in the presence of sodium carbonate and sodium iodide. The most active compound, a *N*10,*O*11-bis-alkylamine indolo[3,2-*b*]quinoline derivative, presented IC_50_ values of 25 and 156 nM for *Pf*W2 and *Pf*3D7, respectively, and LD_50_ values of 39.9 and 23.3 µM for *Pf*Dd2 and *Pf*3D7, respectively. SAR studies showed that lipophilicity and a chlorine at C3 increased cytostatic and cytocidal activities for this scaffold. Moreover, the compounds bind to hematin monomer, inhibiting β-hematin formation and delaying intraerythrocytic parasite development [65].

Following this work, the same authors developed a series of *N*10,*N*11-di-alkylamine bioisosteres (Scheme 2), with the rationale that replacement of the oxygen atom by a NH group would increase the basicity of the quinoline nitrogen, leading to improved accumulation in the digestive vacuole by a pH-trap mechanism. Results showed that the compounds exhibited selectivity for malaria parasite compared to human cells, with IC_50_ values against *P. falciparum* W2 strain in the nanomolar range, and showed improved vacuolar accumulation ratios [66].

**Scheme 3:**
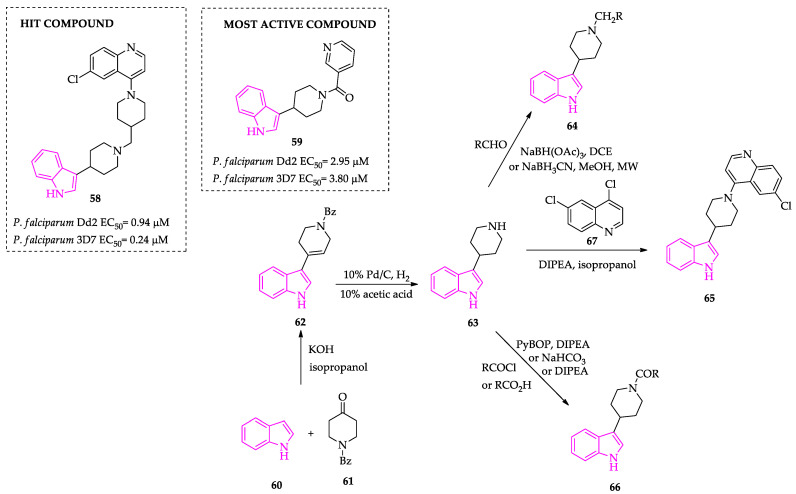
Synthesis of 3-piperidin-4-yl-1*H*-indoles.

In 2015, the same authors developed 38 3-piperidin-4-yl-1*H*-indoles to be tested as antimalarials, based on the hit TCMDC-134281 that emerged in the GSK HTS whole-cell screen against *P. falciparum* to identify novel indole-based antimalarials. Condensation of indole with *N*-benzyl-4-piperidone in the presence of KOH, followed by debenzylation and olefin reduction, afforded the common intermediate 3-piperidin-4-yl-1*H*-indole (Scheme 3). Reductive amination of the amine intermediate with the corresponding aldehyde led to the synthesis of compounds with *N*-piperidinyl modifications. The amide derivatives, prepared to evaluate the importance of the basic amine versus an amide linkage, were prepared by coupling the amine intermediate with acyl chlorides/carboxylic acids or through a nucleophilic substitution with 4,6-dichloroquinoline (**67**). The SAR study showed that the 4-aminoquinolinyl moiety can be replaced by smaller groups without much of an effect on the activity. Only three compounds were active, and of these, only one showed antimalarial activity against drug-resistant and sensitive strains, selectivity for malaria parasite and no cross-resistance with chloroquine [67].

**Figure 6:**
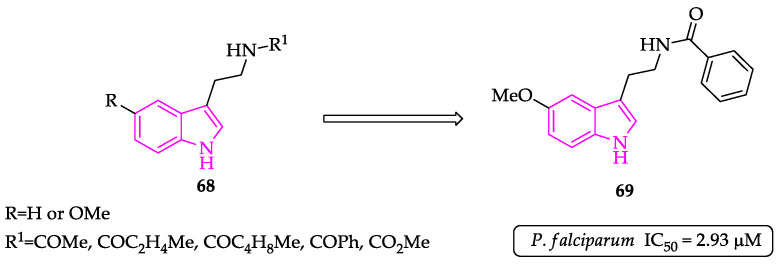
Melatonin derivatives tested against *P. falciparum*.

Melatonin analogs **68** were tested against 3D7 *P. falciparum* strain. The results showed that compounds with a hydrogen instead of a methoxy (R substituent) and with chain elongation of the amino group lead to a decrease *P. falciparum* cell cycle modulation. Compound **69** inhibited the *P. falciparum* growth in the low micromolar range, displaying an IC_50_ value of 2.93 µM (Figure 6) [68].

**Figure 7:**
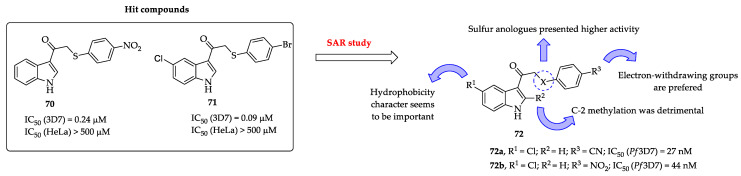
SAR study of indolyl-3-ethanone ethers and thioethers.

To expand a previous SAR study regarding a novel class of indole-based compounds with promising antimalarial activity (representative compounds **70** and **71**, Figure 7), Lunga et al. designed a library of 20 derivatives, using a bioisosteric replacement approach and combinations of promising substituents. Results showed that the replacement of the thiocarbonyl group with an α-oxo carbonyl group was well tolerated, although the sulfur analogs exhibited superior performance in terms of parasite growth inhibition. Another structural trend that proved to be beneficial in terms of activity was the presence of electron-withdrawing groups at the phenyl ring substituent (although it seemed to have size restriction). Regarding the C-5 substitution, hydrophobicity appears to be more significant than the electron-withdrawing nature, with chlorine atom generating the best results. From this study, Lunga et al. identified two analogs, **72a** and **72b**, with IC_50_ values in the low nanomolar range against 3D7 *P. falciparum* strain. In addition, these compounds proved to be nonhemolytic and nontoxic against the HeLa cell line [69].

**Figure 8:**
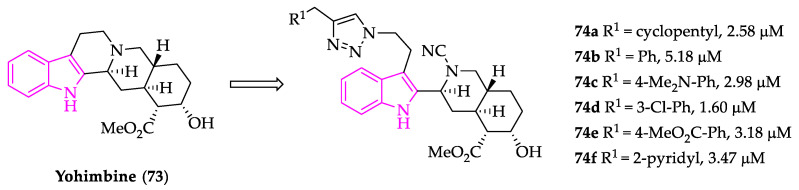
Yohimbine (**73**) ring distortion led to analogs active against Dd2.

Re-engineering of the indole alkaloid yohimbine (**73**) through ring rearrangement and ring cleavage synthesis pathways led to a new class of antiplasmodial agents (Figure 8). The most active compound (**74d**) presented an IC_50_ of 1.60 µM against chloroquine-resistant *P. falciparum* Dd2 strain and was selective over HepG2 cells (CC_50_ = 18.9 µM) [70].

**Scheme 4:**
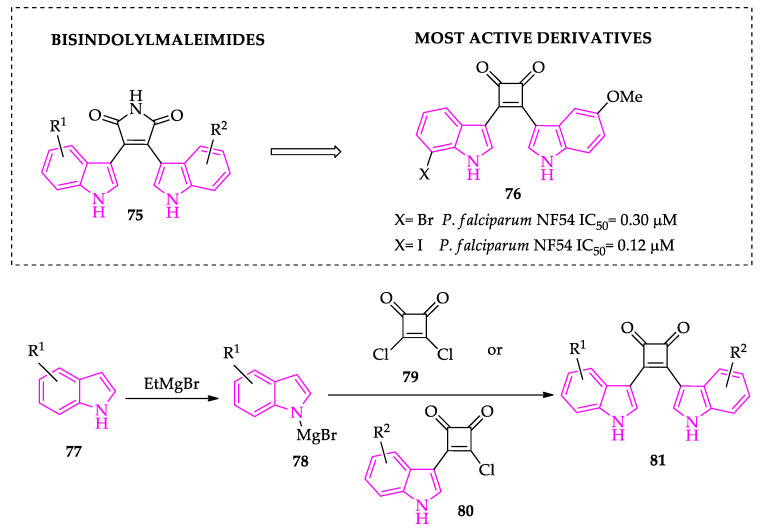
Synthesis of 3,4-bis(indol-3-yl)cyclobut-3-ene-1,2-diones.

More recently, a series of bisindolylcyclobutenediones (Scheme 4), analogs of the bisindolylmaleimide protein kinase inhibitors, were designed in order to obtain plasmodial kinase inhibitors. Based on the results of docking into the ATP binding pockets of some plasmodial protein kinases, some compounds were synthesized and evaluated against transgenic NF54-*luc P. falciparum* parasites. Most derivatives showed submicromolar activity, and some displayed some selectivity against the human cell line THP-1. The most active compounds did not inhibit the plasmodial protein kinase PfGSK-3, so further studies are required to elucidate the mechanism of action of these compounds and to design derivatives with optimized aqueous solubility [71].

**Figure 9:**
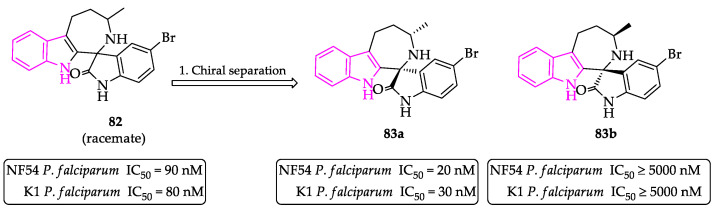
Activities of racemic spiroazepineindole **82** and individual stereoisomers.

NITD609 was obtained by optimization of a lead compound identified by a high-throughput screening of approximately 12,000 compounds. From this screening emerged a racemic spiroazepineindole (compound **82**), an inhibitor of asexual blood-stage *P. falciparum* growth, with IC_50_ values of 90 and 80 nM against wild-type (NF54) and chloroquine-resistant (K1) strains, respectively. A single 100 mg/kg dose of compound **82** led to a reduction of 96% in parasitemia in a *P. berghei* infected mouse model [72]. The individual enantiomers were isolated by chiral separation and tested in vitro. Biological evaluation showed that enantiomer 1*R*,3*S* (compound **83a**) is 250-fold more potent than enantiomer 1*S*,3*R* (compound **83b**) (Figure 9) [73].

**Scheme 5:**
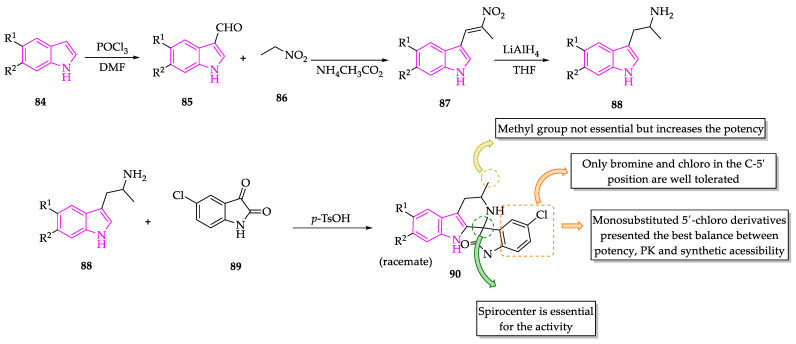
Synthesis of spirotetrahydro β-carbolines.

To increase potency and in vivo activity, a series of spirotetrahydro β-carbolines were also prepared. The synthesis involved Vilsmeier–Haack formylation of the indole, followed by condensation with nitroethane, which led to the corresponding nitroalkene that was reduced to the corresponding amine [73]. Condensation of the indoleamine **88** with 5-chloroisatin (**89**) led to the target racemic products **90** (Scheme 5). The diastereomers were isolated by chiral chromatography and tested individually, and it was also observed that the 1*R*,3*S* configuration was the required stereochemistry for activity against *P. falciparum*. In the SAR study with spiroazepineindoles and spirotetrahydro β-carbolines, it was also concluded that (i) the spirocenter is an essential feature for activity; (ii) the only halogens well tolerated at position 5′ are chloro and bromide; (iii) monosubstituted 5′-chloro derivatives have the optimum balance between potency, favorable pharmacokinetics and synthetic accessibility compared with other mono- and disubstituted oxindole derivatives prepared; and (iv) increasing the size of the central ring azepine ring by one carbon leads to an inactive compound. Interestingly, while the C-3 methyl does not seem to be essential for activity in the azepineindoles, it enhances potency in the tetrahydro β-carbolines [73].

**Figure 10:**
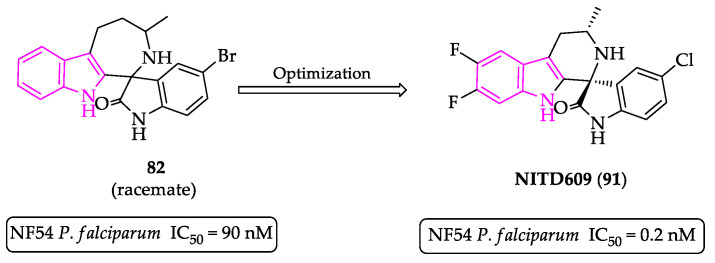
Optimization of compound **82**.

The active enantiomer that displayed subnanomolar potency and possessed better metabolic stability was NITD609 (**91**) (also known as KAE609 and cipargamin, Figure 10), with both C6 and C7 substitutions. NITD609 showed excellent in vitro potency, good bioavailability, long oral exposure, a half-life of 3 h and excellent in vivo activity in a *P. berghei* malaria mouse model. NITD609 was active against *P. falciparum* and *P. vivax* with IC_50_ in the low nanomolar range, dysregulating the sodium and osmotic homeostasis of *Plasmodium* through inhibition of PfATP4, while not displaying significant cytotoxicity against mammalian cells [73,74,75].

Most indole-based compounds with antiplasmodial activity are natural-based compounds (e.g., indole alkaloid cryptolepine) and exert their activity against chloroquine-resistant *Plasmodium* strains by an unknown mechanism of action or by inhibiting hemozoin formation [59,60]. An exception is the spiroindolone NITD609; reported in 2010, it is the first molecule with a novel mechanism of action to successfully complete phase II studies for malaria in the last 25 years [76].

### 2.2. Indole Derivatives with Antitrypanosomal Activity

**Figure 11:**
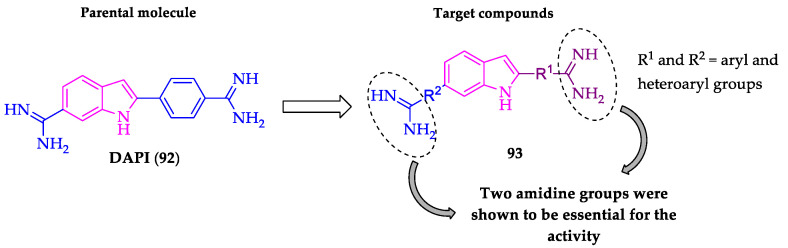
SAR for DAPI analogs **93**.

Farahat et al. reported the synthesis of a library of diamidine indole derivatives (Figure 11) which were evaluated for their antiparasitic activity against *T. brucei* and *P. falciparum*. The target compounds were designed based on the antitrypanosomal activity of the fluorescent dye DAPI (**92**). Most of the indole diamidines exhibited excellent inhibitory activity, with IC_50_ values in nanomolar range for both parasites, and presented superior selectivity index in comparison to the parental molecule DAPI and other diamidines in clinical use, such as furamidine and pentamidine. A structural feature that proved to be essential for activity was the presence of two amidine groups in the compounds [77,78,79].

**Figure 12:**
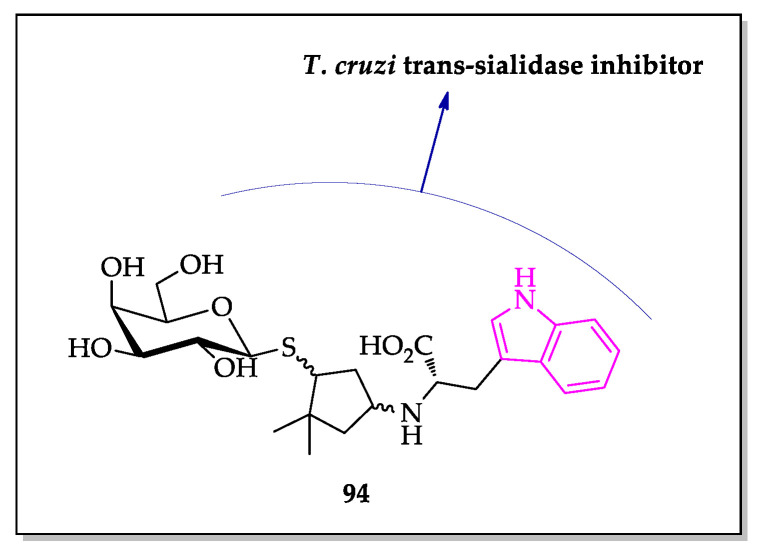
Example of an indole-based galactoside with inhibitory activity of *T. cruzi* trans-sialidase.

In a screening of inhibitors and substrates for *T. cruzi* trans-sialidase (TcTS), Harrison et al. identified that the presence of an indole in the structure of galactosides could be an advantageous feature for the development of TcTS inhibitors. In particular, the thioglycopyranoside **94** ranked among the top five inhibitors in this study (Figure 12) [80].

**Figure 13:**
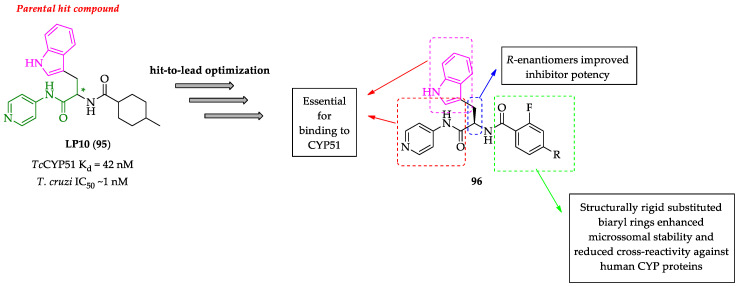
SAR for indolylpirimidinecarboximide-based *Tc*CYP51 inhibitors.

Another successful example of a *Trypanosoma* enzyme inhibitor containing an indole moiety is the *N*-[4-pyridyl]-formamide LP10 (**95**) (Figure 13), a non-azolic sterol 14α-demethylase (CYP51) inhibitor. This compound exhibited high binding affinity for the *T. cruzi* CYP51 (*Tc*CYP51) (K*d* = 42 nM) and high trypanocidal activity (IC_50_ lower than 1 nM) along with low cytotoxicity. Importantly, the indole ring appeared to be an essential structural feature for interactions with the enzyme. Due to pharmacokinetic problems, such as low stability in liver microsome and poor selectivity against the human cytochrome P450 enzymes, an extensive hit-to-lead optimization of compound **95** was developed. The results showed that *R*-enantiomers have increased potency for the target enzyme [81,82,83,84,85] and that the replacement of the cyclohexane ring with structurally rigid substituted biphenyl rings could enhance pharmacokinetic properties while reducing cross-reactivity against human CYP proteins [81,82,83,84,85,86].

**Figure 14:**
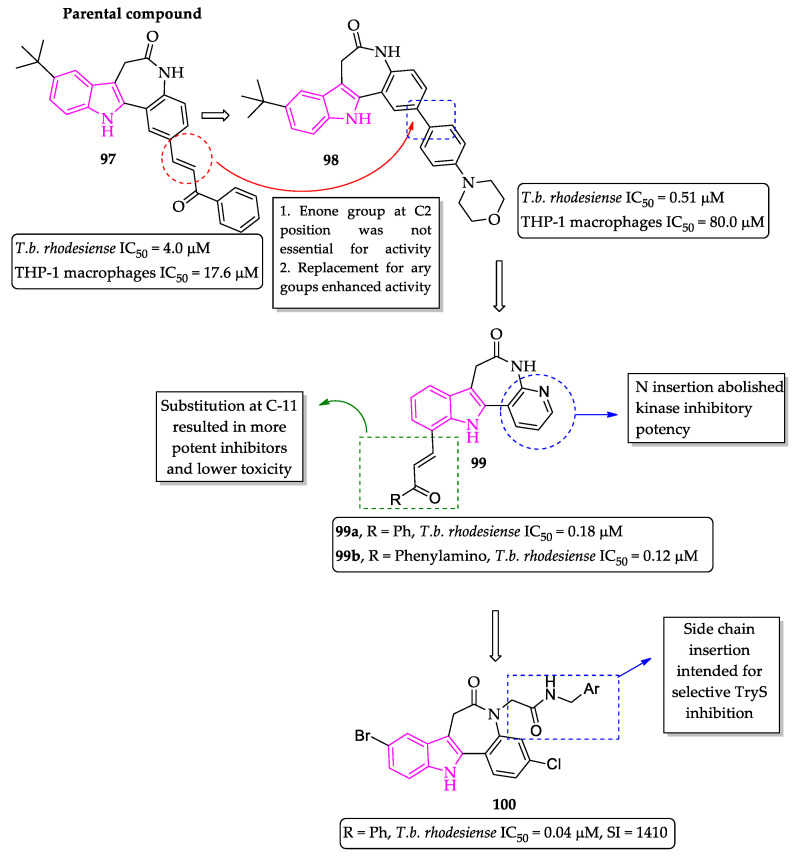
Paullone scaffold optimization.

Inspired by the results of the antileishmanial activity with synthetic paullone–chalcone hybrids, Kunick and collaborators decided to extend the study for *T. brucei* parasites (Figure 14). Initially, the authors investigated the influence of Michael acceptor groups at position C2 of the paullone ring system on the selectivity of these compounds, since α,β-unsaturated electrophilic groups can lead to the formation of Michael adducts with host biomolecules and undesired toxicity. From this screening, they were able to identify that the enone group at this position was irrelevant for inhibitory activity on parasite growth, while the replacement with an aryl group improved the cytotoxic profile of the compounds (compound **97** IC_50_ = 4.0 µM and compound **98** IC_50_ = 0.51 µM). Part of the toxicity of these compounds could be attributed to host protein kinase inhibition, and therefore, the authors continued the optimization process by synthesizing 4-azapaullone derivatives substituted at C-9 and C-11 positions (Figure 14). The new generated 4-azapaullone derivatives retained antiparasitic properties and exhibited limited cytotoxicity against mammalian cells. Particularly, compounds with phenyl or phenyl-amino substituent at position C-11 showed improved inhibitory profile (compound **99a** IC_50_ = 0.18 µM and compound **99b** IC_50_ = 0.12 µM). In addition, the most active compounds failed to inhibit a panel of mammal kinases. Finally, following a structure-based rational design to target the enzyme trypanothione synthetase (TryS), the same research group developed a new series of *N*(5)-substituted paullones containing benzyl or heteroarylmethyl substituents. Although the new compounds showed potent and selective trypanocidal activity (compound **100** IC_50_ = 40 nM) on infective forms of *T. brucei*, they exhibited only a modest improvement over the template molecule in the inhibitory activity towards the TryS enzyme, suggesting a possible different mechanism of action [87,88,89].

**Figure 15:**
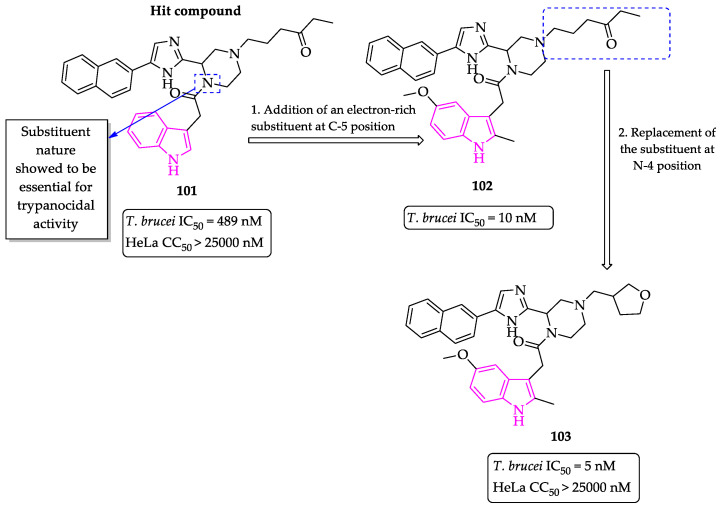
Optimization of compound **101**.

Aiming to develop potent *T. brucei* histone deacetylase (HDAC) inhibitors, Ferrigno et al. reported the synthesis and optimization of 2,5-disubstituted imidazole-based compounds, bearing a naphthyl group at position 5 and a 2-piperazinyl ring at position 2 [90]. The initial SAR study identified the importance of the substituent at the N-1 position of the piperazine ring for the trypanocidal activity, and the electron-rich 2-methyl-5-methoxy indole group was responsible for the best activity profile in the first set of compounds. Further steps of optimization led to the discovery of an inhibitor (**103**) that exhibited strong trypanocidal activity in the single-digit nanomolar range (Figure 15). However, the compound exhibited only a modest half-life in presence of human and mouse hepatocytes, indicating the need for further modifications aiming at better pharmacokinetic properties [90].

**Scheme 6:**
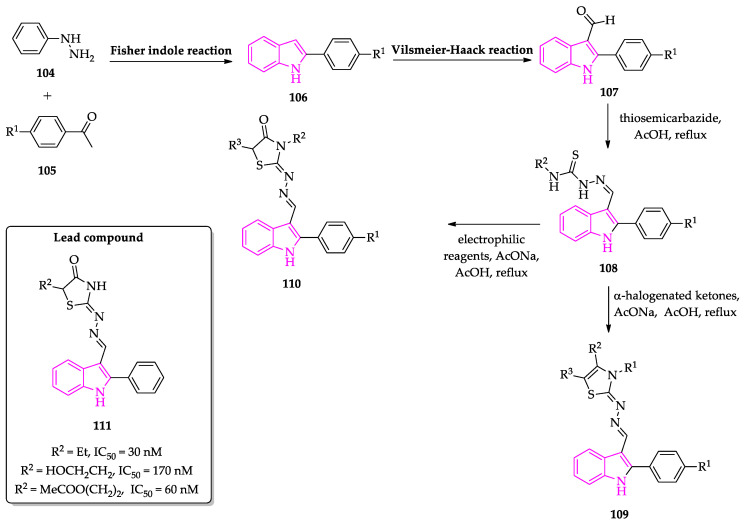
Synthesis of 4-thiazolidone and thiazole phenyl-indole derivatives.

Using a molecular hybridization approach, Kryshchyshyn et al. generated a diverse library of compounds bearing 4-thiazolidone or thiazole group tethered by a hydrazone linker to a phenyl-indole or phenyl-imidazothiadiazole fragment. The synthesis of phenyl-indole compounds bearing thiosemicarbazones was accomplished in three steps: (1) reaction of phenylhydrazine with substituted aryl methyl ketones, *via* Fisher indole method; (2) Vilsmeier–Haack reaction to generate the indole-3-carbaldehyde derivative (**107**); (3) condensation with thiosemicarbazides (Scheme 6). Then, thiazolidinone–indole hybrids were obtained by [2+3]-cyclocondensation reaction with different electrophilic reagents. The thiazole-hydrazone analog was synthetized by reaction with α-halogenated ketones [91]. This chemical library was screened against *T. brucei*, and compounds with submicromolar IC_50_ values and low cytotoxicity in human primary fibroblasts were identified. The SAR study evidenced that the indole scaffold was essential for the inhibitory activity as 2-arylindole-based compounds (**109**–**110**) were more active than the 6-aryl-imidazo[2,1-*b*][1,3,4]thiadiazole counterparts [91].

**Scheme 7:**
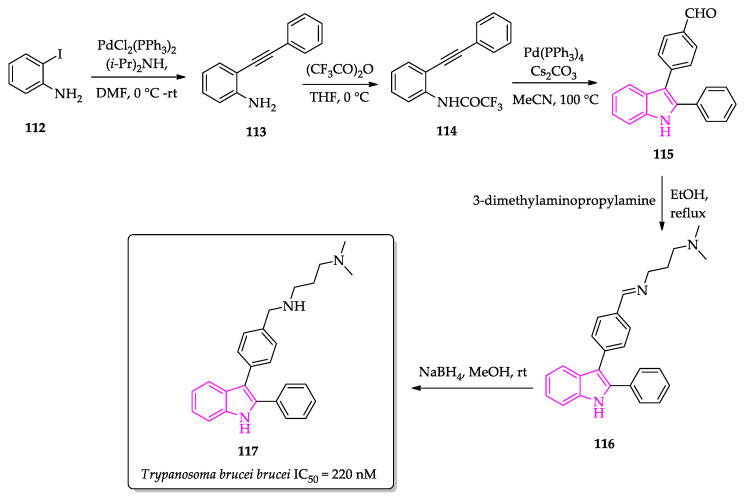
Synthetic route to obtain 1*H*-3-{4-[(3-dimethylaminopropyl)aminomethyl]phenyl}-2-phenylindole (**117**).

Working on the development of antimalarial compounds containing an indole moiety, Guillon et al. reported the trypanocidal activity of another 2-phenylindole derivative. The synthesis started with the preparation of *o*-(phenylethynyl)aniline by a Sonogashira cross-coupling reaction between commercially available *o*-iodoaniline and phenylacetylene, which was converted to *o*-alkynyltrifluoroacetanilide upon reaction with trifluoroacetic anhydride (Scheme 7). In sequence, the 2-phenylindole scaffold was obtained by an aminopalladation–reductive elimination protocol, yielding the aldehyde **115** [92,93]. Finally, the target compound **117** was obtained by condensation of **115** with 3-dimethylaminopropylamine followed by reduction with sodium borohydride [93]. The compound exhibited antiparasitic activity against all evaluated protozoa with moderate cytotoxicity towards the human cell line HepG2. However, in the case of *T. b. brucei* (IC_50_ = 220 nM), the selectivity index showed reasonable value, which indicates that compound **117** may be valuable for future optimizations [93]. 

**Scheme 8:**
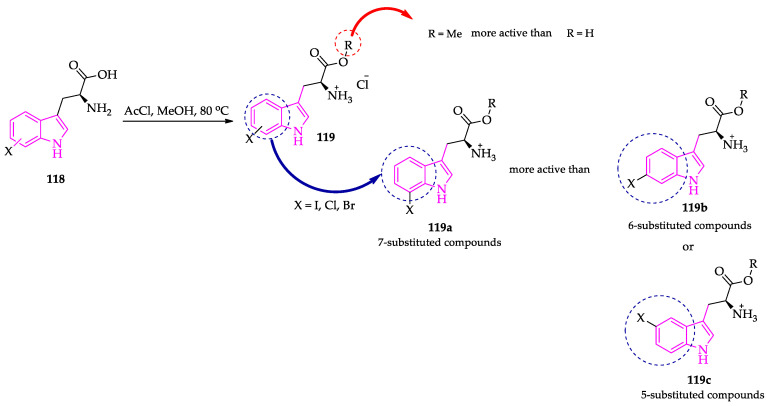
Halogenated *L*-tryptophan analogs **119** with trypanocidal activity against *T. brucei* parasites.

Considering that the particular amino acid metabolism by trypanosomatid parasites, especially *T. brucei*, may be a target for the design of new and selective inhibitors, Cockram et al. investigated the trypanocidal activity of halo-substituted *L*-tryptophan free acids and methyl ether derivatives (Scheme 8). In general, the SAR indicated that, regardless of the halogen atom, the 7-substituted compounds exhibited significantly higher potency compared to the 5- and 6-substituted counterparts. Except for natural *L*-tryptophan, methyl esterification also increased the activity of the compounds. The authors also identified that these analogs exhibited a competitive relationship with natural tryptophan. Although the metabolomic studies suggested an effect on the transamination processes of the parasite, the authors were unable to determine the exact mechanism of action of the compounds [94].

**Figure 16:**
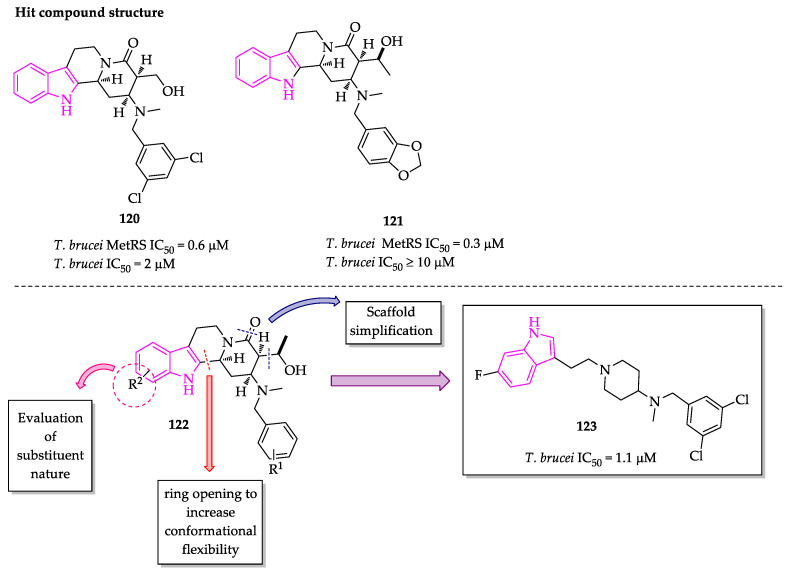
Rationale for structure-based design of novel hydroxyalkyl δ-lactam, δ-lactam and piperidine indole derivatives.

Inspired by the two indole-based *Yohimbine* and *Corynanthe* alkaloids, Lepovitz et al. generated a diverse series of hydroxyalkyl δ-lactam, δ-lactam and piperidine indole derivatives, following a structure-based rational design of small-molecule inhibitors for the *T. brucei* methionyl-tRNA synthetase (TbMetRS) (Figure 16). Most compounds exhibited a good trypanocidal profile, although there was no significant improvement in the potency of TbMetRS inhibition in relation to the hit compound, which suggests an alternative mechanism of action [95].

**Scheme 9:**
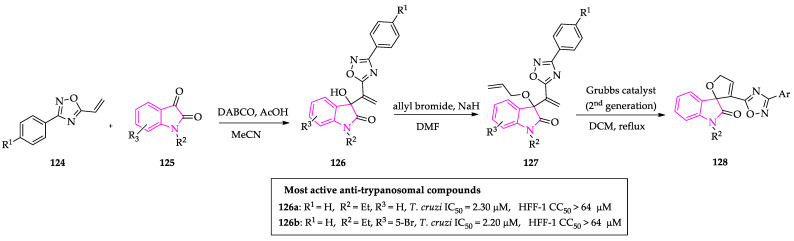
Synthesis and antitrypanosomal activity of oxadiazole–oxindole hybrids.

Fernandes et al. envisioned a molecular hybridization approach to unite two known bioactive heterocyclic scaffolds, oxadiazoles and 3-hydroxy-2-oxindoles, to generate new antiparasitic compounds (Scheme 9). To achieve this purpose, the authors performed a Morita–Baylis–Hillman (MBH) reaction between 3-phenyl-5-vinyl-1,2,4-oxadiazole derivatives and *N*-ethyl-isatin in the presence of DABCO and acetic acid, obtaining 3-hydroxy-2-oxindoles (Scheme 9). In addition, they also synthetized two spirooxindole derivatives using a two-step procedure: *O*-allylation followed by ring-closing metathesis. Interestingly, some of the hybrid compounds exhibited excellent antiparasitic activity (IC_50_ = 2.30 µM and 2.20 µM) and a low cytotoxic profile against human fibroblasts (CC_50_ > 64 µM). Moreover, some of them also presented efficacy values similar or superior to benznidazole [96].

**Figure 17:**
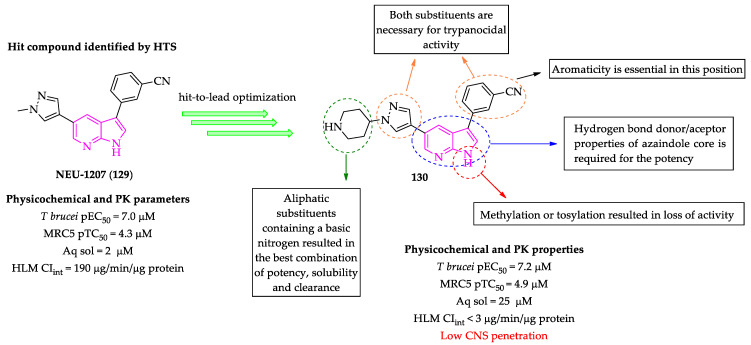
Hit-to-lead optimization of 3,5-disubstituted-7-azaindole NEU-1207 (**129**).

By a high-throughput screening over a large library of kinase inhibitors, using as a filter the trypanocidal activity, low toxicity over mammal cells and other physicochemical properties, Klug et al. selected the 3,5-disubstituted-7-azaindole (**129**) (NEU-1207) as hit compound for further structural optimization, aiming to improve inhibitory activity and pharmacokinetic properties (Figure 17). Using a very detailed SAR, the authors identified compound (**130**) that presented both submicromolar potency against *T. brucei* and an adequate pharmacokinetic profile. However, the compound also presented a low potential to penetrate the central nervous system, which would prevent it from acting as an effective antitrypanosomal agent in stage 2 of HAT [97].

### 2.3. Indole Derivatives with Antileishmanial Activity

Drug discovery for leishmaniasis treatment has also suffered from slow progress, with few drug candidates entering clinical trials in recent years [98,99]. Besides problems with toxicity and low efficacy of the available treatments, the emergence of drug resistance has further complicated the disease management scenario [100]. Thus, the identification of new chemotypes plays an essential role in addressing these urgent issues.

**Scheme 10:**
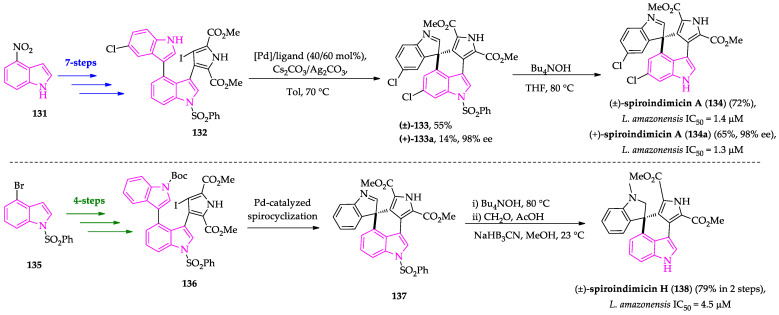
Summary of the total synthesis of spiroindimicins A (**134**) and H (**138**).

Through a Pd-catalyzed asymmetric spirocyclization approach, Zhang et al. achieved the intricate task of preparing the spiroindimicins A and H and analogs in a gram scale (Scheme 10). The synthesis of spiroindimicin A (**134**) involved a nine-step route starting from the commercially available 4-nitroindole (**131**), which was converted to the key triaryl intermediate (**132**), followed by a Pd-catalyzed spirocyclization and removal of a benzenesulfonyl group with Bu_4_NOH [101]. Using the appropriate combination of chiral ligand, bases and reaction conditions, the authors were able to obtain the desired product in moderate yields and with high enantioselectivity. With this optimized methodology, they also achieved the synthesis of spiroindimicin H (**138**) from 4-bromoindole (**135**) in eight steps (Scheme 10) [101]. The final compounds, as well as some intermediates, were evaluated against *T. brucei*, *P. falciparum* and *L. amazonensis*. Several compounds presented good inhibitory activity against the different parasites, particularly spiroindimicin A, which showed IC_50_ = 1.3 µM with minimal toxicity for mammal HepG2 and RAW 264.7 cells (CC_50_ > 10 µM) [101].

**Scheme 11:**
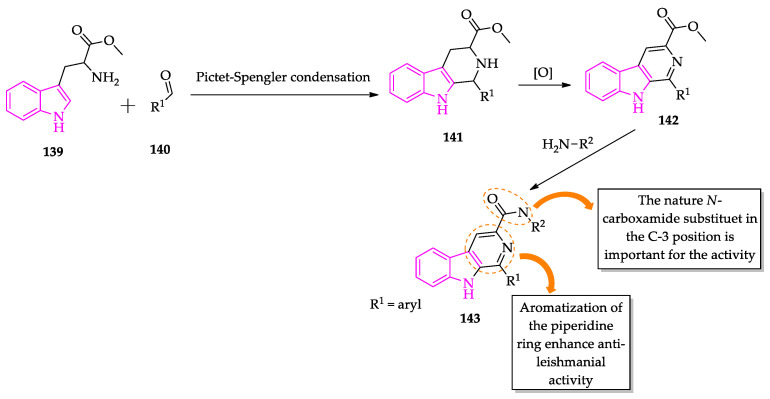
Synthesis of β-carboline-3-carboxamides with antileishmanial activity.

Over the years, synthetic compounds inspired by indole alkaloids have been investigated for their antileishmanial potential, mainly due to evidence of the inhibitory properties of this class against other trypanosomatid parasites. One example is the β-carboline family, which has shown inhibitory activity against both *T. cruzi* and *Leishmania* spp. parasites. Working with β-carboline-3-carboxamide derivatives, Tonin et al. studied the influence of the piperidine ring oxidation on the antiprotozoal activity. The target compounds were prepared by a Pictet–Spengler condensation of the *L*-tryptophan methyl ester with aromatic aldehydes, followed by oxidation with sulfur in xylene reflux (Scheme 11). Subsequently, the corresponding carboxylates were reacted with different amines to generate the desired carboxamides. The compounds were evaluated in vitro against the epimastigote form of *T. cruzi* and the promastigote form of *L. amazonensis*. The authors observed that 1,2,3,4-tetrahydro-β-carboline exhibited lower potency than the corresponding oxidized counterparts, indicating that ring aromatization is an important structural feature regarding the biological activity [102]. In a subsequent study with axenic amastigote forms, it was also possible to identify the relevance of the nature of the substituent at the C-3 of the β-carboline nucleus. In general, this type of compounds exhibited low to moderate cytotoxicity and high selectivity towards parasite cells, indicating that it is indeed a promising scaffold for further optimizations [103].

**Figure 18:**
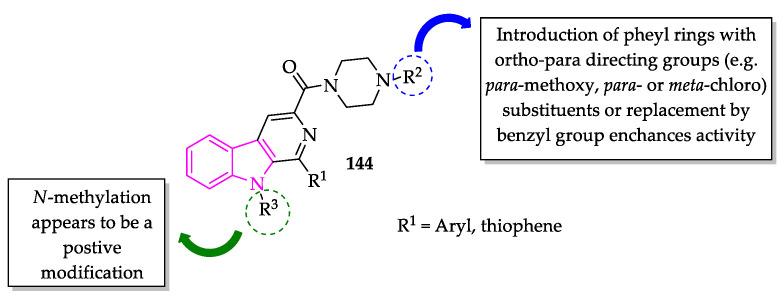
General structure of piperazinyl-β-carboline-carboxamides with antileishmanial activity.

Keeping the carboxamide group at the C-3 position, Ashok et al. designed a series of piperazinyl-β-carboline hybrids, bearing a thiophene ring in the C-1 position of the pyridine ring (Figure 18) [104]. They generated a small library of 15 compounds, changing the nature and position of the substituent of the aromatic ring attached to the piperazine group. With this approach, the authors were able to map the most relevant structural characteristics for the activity. The first conclusion was that an unsubstituted ring resulted in very weak potency against promastigote parasites, while *para*-substitution with small and *ortho–para* directing groups generated compounds with high antileishmanial activity. Substitution in other positions (e.g., 2-methoxy or 3-methoxy) produced an increase in growth inhibition, as well as cytotoxicity. Replacement with a benzyl group could also generate a positive effect in potency with a moderate selectivity index. Some of these compounds also showed high activity against intracellular amastigote forms [104]. In a subsequent SAR study with *N*-methylated beta-carboline-3-carboxamide compounds, the same research group was able to corroborate the substitution patterns of the piperazinyl group that can impact the activity of this family. Some compounds containing aromatic rings with *para*-methoxy, *para*- or *meta*-chloro or even the replacement with benzyl group presented strong activity against both promastigote and amastigote forms of *L. donovani* and *L. infantum*, with high selectivity against parasite cells [105]. From this work, it is possible to assume that *N*-protection of indole moiety can also be a relevant modification [105].

**Figure 19:**
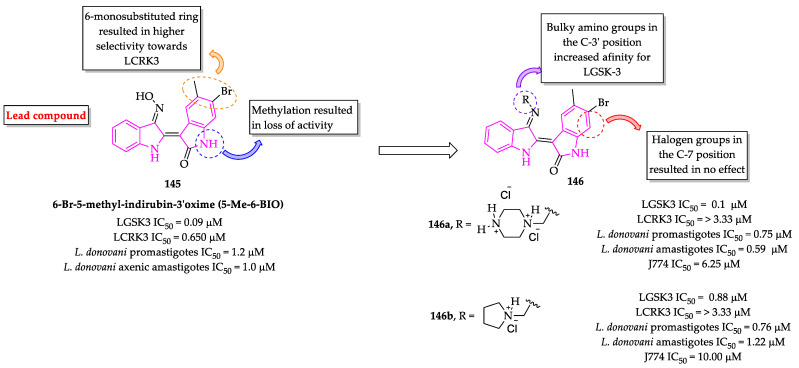
Structure–activity relationship of indirubin analogs for the LGSK3 enzyme.

Indirubins are another example of indole alkaloids that have been investigated for their antileishmanial properties. Naturally, this class of bis-indole compounds is found in different living organisms such as indigo-bearing plants (e.g., *Isatis* spp. and *Polygonum* spp.), marine mollusks and bacteria [106,107]. It has been identified that indirubins are potent ATP-competitive inhibitors of protein kinases (CDKs) and glycogen synthase kinase 3 (GSK-3β) [108]. In addition, it has been shown that 6-substituted indirubin derivatives could inhibit both leishmanial cyclin-dependent kinase 1 (CDK1) homologs, cdc2-related protein kinase 3 (LCRK3) and glycogen synthase kinase 3 (LGSK-3), two enzymes essential for parasite viability [109]. Interestingly, a higher selectivity towards LCRK3 over LGSK was further observed, a reverse selectivity in comparison to the mammalian counterparts [109]. Thus, Efstathiou et al. performed an SAR study over a library of indirubin derivatives to identify structural features that are necessary to change the selectivity pattern of these two leishmanial enzymes. From this study, some structural assumptions could be made regarding selectivity towards LGSK-3 and the protein binding mode of these compounds (Figure 19). First, it was identified that the presence of halogen groups in the position C-7 had no significant effect on activity against both promastigote and amastigote forms. In addition, it was observed that the introduction of *N*-containing saturated ring in the position C-3′ was sufficient to reverse the selectivity towards LGSK-3, probably by increasing the affinity of the compounds for hydrophilic sites of the binding pocket, which could result in the displacement of unstable water molecules from the kinase hydration shell [110].

**Scheme 12:**
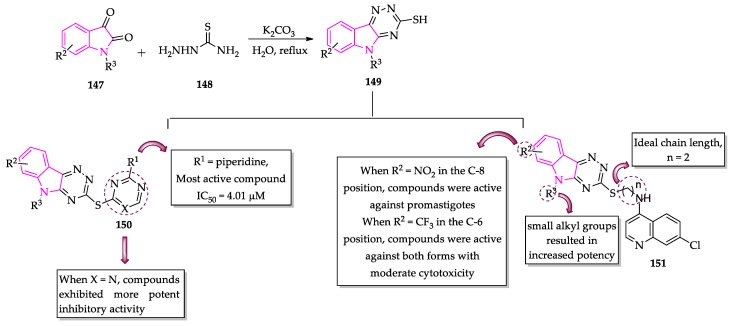
Triazino[5,6-*b*]indole hybrids with antileishmanial activity.

Aiming to obtain new inhibitors of dihydrofolate reductase (DHFR), another enzyme important for the parasite survival, Chauhan’s group planned a molecular hybridization approach, joining a 1,3,5-triazine or pyridine unit to the [1,2,4]triazino[5,6-*b*] indole moiety (Scheme 12). This strategy resulted in 22 hybrids that were subjected to in vitro biological evaluation against *L. donovani* parasites [111]. The results showed triazine derivatives (**150**) were more potent than the corresponding pyrimidine analogs, particularly the triazine derivative with R^1^ = piperidine (*L. donovani* IC_50_ = 4.01 µM, J774.A1 CC_50_ = of 227.04 µM), which was 20- to 10-fold more selective than standard drugs such as pentamidine and sodium stibogluconate [111]. Subsequently, the same research group developed a series of [1,2,4]triazino[5,6-*b*] indole hybrids (**151**) with 4-amino-7-chloroquinoline. Changing the linker size, the protection group attached to the nitrogen atom of the indole moiety and the nature of substituent in the position C-6 or C-8 of the aromatic ring, the authors were able to identify some lead compounds with high antileishmanial activity and low cytotoxicity. It also was observed that compounds with a linker size of two carbon atoms and with a small alkyl group at nitrogen atom or unsubstituted aromatic ring produced a very potent hybrid for both promastigotes and intracellular amastigotes [112].

**Scheme 13:**
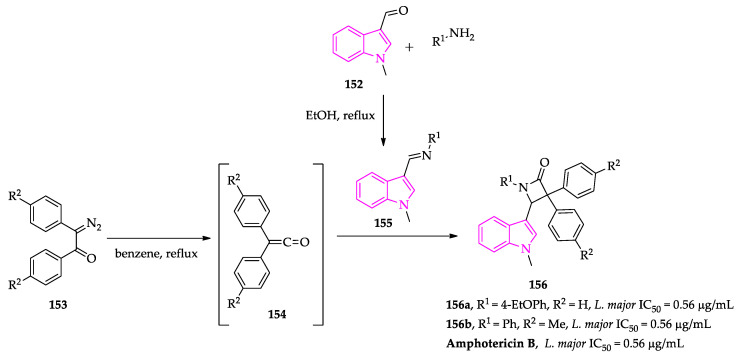
Indole-based azetidin-2-ones with antileishmanial activity.

The association of indole with β-lactam ring also produced compounds with potent antileishmanial activity. These compounds were synthetized by reaction of *N*-(1-methyl-1*H*-indol-3-yl)methyleneamines with 2-diazo-1,2-diarylethanones (Scheme 13). Both indole-based imines and azetidin-2-ones were evaluated against *L. major* promastigotes. Compared to imine precursors **155**, the azetidin-2-one derivatives **156** showed considerable improvement of activity, and two compounds exhibited inhibitory activity in the same range as that of the standard drug amphotericin B [113].

**Scheme 14:**
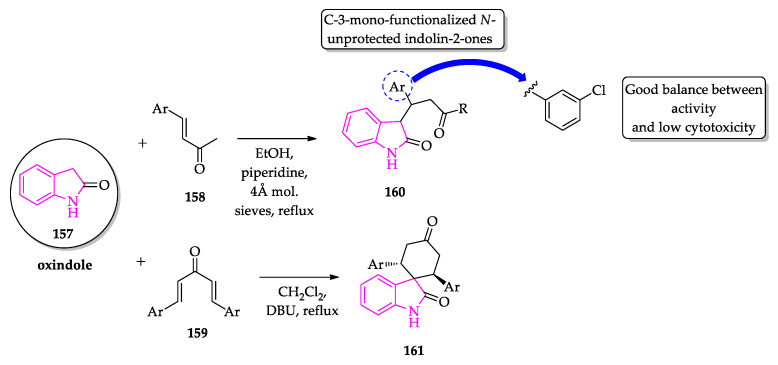
Synthesis of C-3-mono-functionalized *N*-unprotected indolin-2-ones.

Different types of spiro-oxindole derivatives have been synthetized and evaluated against *Leishmania* spp. parasites, showing potent inhibitory activity. Scala et al. reported a simple and fast synthetic way to functionalize the C-3 position of *N*-unprotected oxindoles (Scheme 14). Using dibenzalacetones as reactants and 1,8-diazabicyclo[5.4.0]undec-7-ene (DBU) as base catalyst, the authors achieved the synthesis of spiro[cyclohexanone-oxindoles] **161**. In general, the evaluation of antileishmanial activity revealed that both oxindole precursors and spirooxindole derivatives exhibited significant dose-dependent inhibitory activity against promastigote and amastigote forms of *L. infantum*, low cytotoxic profile against RAW 264.7 cells and high therapeutic index [114].

**Scheme 15:**
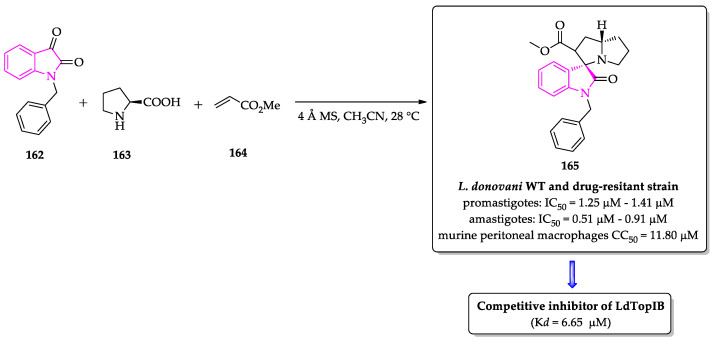
Synthesis of spirooxindole (**165**), a possible competitive inhibitor of LdTopIB enzyme.

Reinforcing the antiparasitic potential of this class of compounds, a small library of spirooxindoles and bis-spirooxindoles was screened against *L. donovani* topoisomerase IB (LdTopIB), another enzyme essential in the maintenance of cellular functioning and a relevant molecular target for antileishmanial therapy [115]. The compounds were synthetized by reaction of isatin (or *N*-benzyl isatin) with *L*-proline (**163**) in the presence of 4 Å molecular sieves, followed by the addition of an appropriate dipolarophile (Scheme 15) [115]. Among 10 compounds, the authors identified one derivative (compound **165**) that exhibited a dose-dependent inhibitory activity with a competitive inhibitor profile, possibly by preventing the formation of the DNA–topoisomerase complex (K*d* = 6.65 µM). Docking models of the compound and the topoisomerase identified two possible binding sites in the small subunit and the hinge region of the large subunit of LdTopIB [115]. Moreover, compound **165** was highly active against promastigote (IC_50_ values ranging between 1.25 and 1.41 µM) and amastigote (IC_50_ values ranging between 0.51 and 0.91 µM) forms of both wild-type and drug-resistant strains of *L. donovani* with low toxicity towards host cells (CC_50_ of 11.80 µM), indicating that this scaffold presents the adequate characteristics to advance for further optimization [115].

**Scheme 16:**
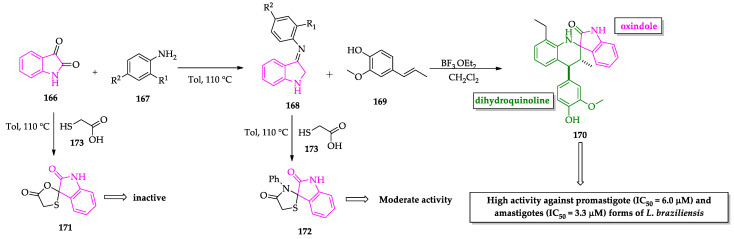
Synthesis of spiro dihydroquinoline-oxindoles with antileishmanial activity.

Another group of spirooxindole derivatives **170**–**172**, containing a dihydroquinoline group, was prepared by an imino Diels–Alder reaction between ketimine-isatins and trans-isoeugenol, using boron trifluoride etherate as acid catalyst [116]. Alternatively, two other spiro compounds were synthetized by a three-component reaction of isatin, aniline and thioglycolic acid and by a two-component reaction of isatin and thioglycolic acid (Scheme 16). Except for only one derivative, most of the evaluated compounds significantly inhibited the proliferation of *L. braziliensis* promastigotes with a very low cytotoxic effect on murine bone marrow-derived macrophages [116]. Particularly, one derivative (**170**, *L. braziliensis* promastigotes IC_50_ = 6.0 μM) exhibited antileishmanial activity superior to the standard drug used as a control in the study (miltefosine, *L. braziliensis* promastigotes IC_50_ = 21 μM) with SI > 50. Comparing the activity of spiro dihydroquinoline-oxindoles **170** with the ones of spiro compounds **171**–**172** prepared from the thioglycolic acid, it was possible to observe that the spiro annulation between quinoline and oxindole backbone was essential for the inhibitory effect on promastigote proliferation. Interestingly, the most active compound also proved to be more potent and selective against intracellular forms of the parasite [116]. The investigation of the mechanism of action showed that this family possibly acts by affecting the activity of the squalene epoxidase enzyme, and consequently the squalene epoxidase enzyme, and/or by disrupting the regulatory volume decrease mechanism [116].

**Scheme 17:**
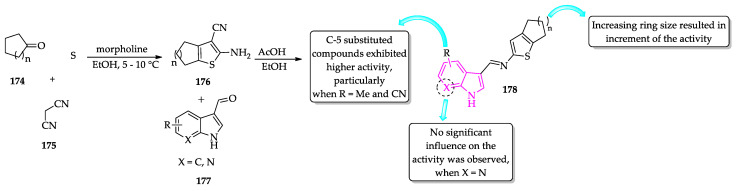
Synthesis and structure–activity relationship study of indole-based 2-amino-thiophene derivatives.

Investigating the antileishmanial potential of the 2-amino-thiophene scaffold, Rodrigues et al. prepared a small library of 2-amino-cycloalkyl[*b*]thiophene-3-carbonitrile derivatives (Scheme 17) [117]. In particular, the examples containing a lateral indole ring exhibited the best results against promastigote and axenic amastigote forms of *L. amazonensis*, causing an apoptosis-like cell death [117]. These compounds exhibited low toxicity against macrophages and red blood cells. Moreover, the selectivity index values were superior to the values calculated for the reference drug meglumine antimoniate [117]. Docking studies identified the trypanothione reductase enzyme as the putative molecular target. In a subsequent study, the authors studied the influence of the cycloalkane size and the nature of the substituent on the indole ring [118]. From 32 evaluated derivatives, they identified 3 other new compounds with a potency superior to reference drugs (IC_50_ values ranging between 2.1 µg/mL and 3.2 µg/mL) and efficacy even against drug-resistant strains [118].

**Scheme 18:**
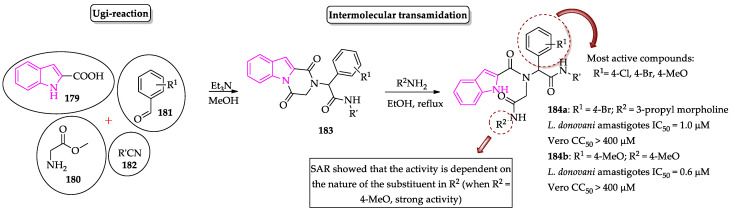
Synthesis of indole-2-carboxamide derivatives via post-Ugi transformation.

Pandey et al. prepared a diversified library of C2-functionalized indole derivatives, using a previously reported protocol of post-Ugi transformation [119,120]. The synthesis of the target compounds was achieved in two steps. First, the multicomponent reaction between 1*H*-indole-2-carboxylic acid (**179**), isocyanide, an amino ester and an aldehyde generated different intermediate indole-fused diketopiperazines. In sequence, the intermediates were refluxed in ethanol with various amines to afford the corresponding carboxamides by a regioselective ring-opening of a diketopiperazine unit by an intermolecular transamidation reaction (Scheme 18). 17 1*H*-indole-2-carboxamides **184** were obtained and their antileishmanial activity evaluated against intracellular amastigote form of *L. donovani* and cytotoxicity on mammal cell line [120]. Most of the compounds exerted antileishmanial activity in the low micromolar range with a potency higher than the standard drug miltefosine [120]. Particularly, two compounds, **184a** (R^1^ = 4-Br, R^2^ = *3*-propyl morpholine) and **184b** (R^1^ = 4-MeO, R^2^ = 4-MeO), displayed low cytotoxic activity (CC_50_ > 400 µM in Vero cell line) and potent inhibitory activity with IC_50_ = 1.0 µM and 0.6 µM, respectively [120]. Similar to what was observed for other indole-based compounds, it seems that this series also exert their antileishmanial activity by induction of an apoptosis-like cell death [120]. In addition, preliminary pharmacokinetic studies in an experimental model indicated that this class of compounds presents good oral bioavailability with no signs of toxicity [120].

**Scheme 19:**
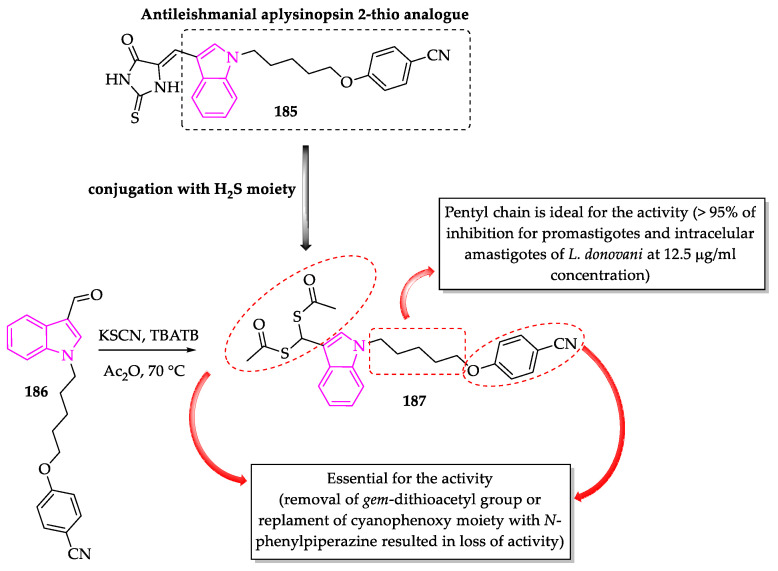
Synthesis and SAR studies of *gem*-dithioacetylated indole.

Having previously identified the antileishmanial activity of a synthetic analog of aplysinopsin (**185**, Scheme 19), Porwal et al. envisioned the conjugation of a H_2_S-donating moiety to enhance the pharmacological properties of this class of compounds [121]. A multicomponent protocol was used to convert 1-alkylated indole-3-carbaldehyde (**186**) into gem-dithioacetylated indole derivatives [121]. Gem-dithioacetyl and cyanophenoxy groups were shown to be important for the antileishmanial activity of these compounds, since the corresponding analogs without these two moieties were revealed to be inactive [121]. Moreover, the size of the linker chain was another structural feature that proved to be quite relevant [121].

**Scheme 20:**
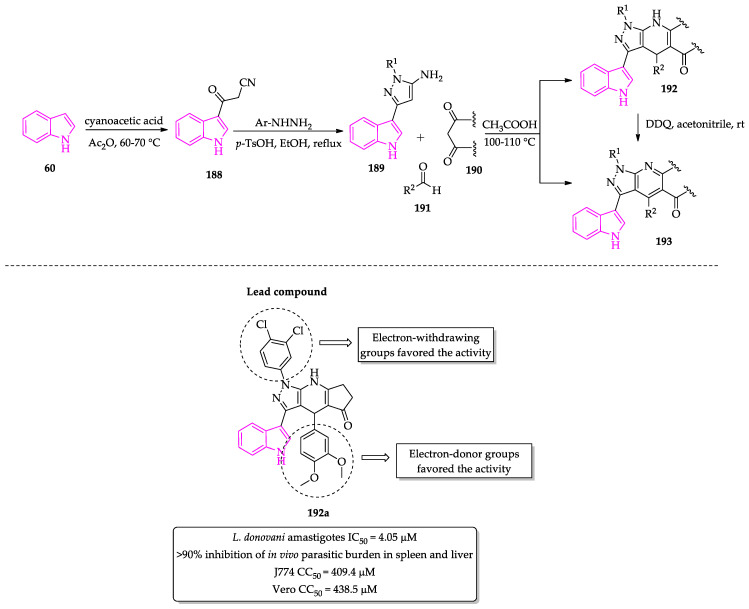
Synthesis of pyrazolodihydropyridine and pyrazolopyridine indole-based derivatives.

By combining an indole scaffold with other pharmacophores commonly found in compounds with antileishmanial activity, Anand et al. designed two series of C3-functionalized indole derivatives, containing pyrazolopyridine and pyrazolodihydropyridine units [122]. These compounds were easily accessed by a multicomponent reaction between 1-aryl-3-indolyl-5-amino pyrazoles **189**, an aldehyde and a 1,3-diketone source (Scheme 20). The in vitro evaluation of the antileishmanial activity revealed two derivatives (**192a**: R^1^ = 3,4-dichlorophenyl, R^2^ = 3,4-dimethoxyphenyl; **192b**: R^1^ = 3,4-dichlorophenyl, R^2^ = 2,5-dimethoxyphenyl) with potent growth inhibition activity against promastigotes and intracellular amastigotes. Moreover, these compounds displayed negligible toxicity against two different mammal cell lines with CC_50_ values > 400 µM. Interestingly, similar SAR trends were observed for both groups of compounds, with those with electron-withdrawing groups (e.g., *p*-chloro, 3,4-dichlorophenyl) on the R^1^ position of the pyrazole ring and electron-donating groups (e.g., 2,5-dimethoxy, 3,4-dimethoxy or 3,4,5-trimethoxyphenyl) on R^2^ of the dihydropyridine or pyridine ring exhibiting the best activity. To corroborate the efficacy and low toxicity of the compounds, the authors also performed in vivo assays and verified that both compounds could reduce parasitic burden in the liver and spleen. In addition, the combination therapy with subcurative concentration of miltefosine allowed a considerable improvement in the disease pattern in murine experimental models [122].

**Scheme 21:**
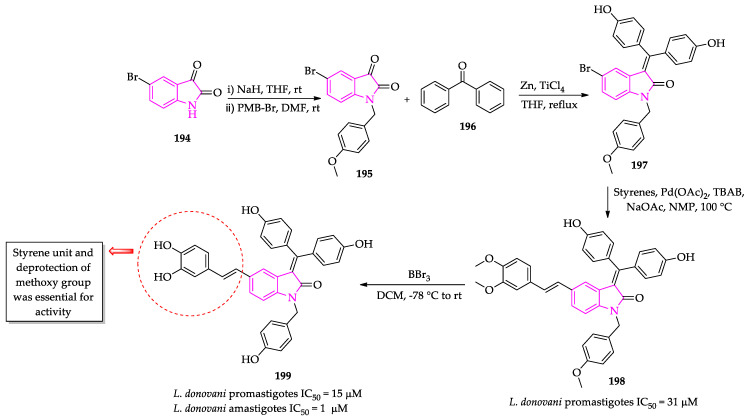
Synthesis of stilbenic oxindole derivatives **199** with antileishmanial activity.

The molecular hybridization of indole nucleus with polyphenolic compounds was shown to be another strategy to obtain derivatives with high pharmacological potential. In this sense, Yousuf et al. reported that the covalent conjugation of an oxindole moiety enhanced the biological activity of bis-phenols and bis-arylidenes [123]. Aiming to develop novel antileishmanial compounds, Yousuf et al. functionalized the C-5 of bis-arylidene oxindoles by the introduction of a styrene unit [124]. The compounds were obtained in four synthetic steps, starting with the preparation of the 5-bromo bis-arylidene oxindole derivative *via* McMurry cross-coupling reaction between 4,4′-dihydroxybenzophenone and *p*-methoxybenzyl-protected 5-bromoisatin in presence of TiCl_4_ (Scheme 21) [124]. The final step was a Heck coupling with different styrene precursors in presence of Pd-catalyst (Scheme 21) [124]. The removal of methoxy groups from the 3,4-dimethoxy styrene conjugated bis-arylidene oxindole (**198**) was performed by treatment with BBr_3_ in dry DCM. Among the group of stilbenic oxindole derivatives, the polyhydroxylated compound (**199**) exhibited the best activity against promastigote (IC_50_ = 15 µM) and amastigote (IC_50_ = 1 µM) forms of *L. donovani*, with negligible toxicity towards murine splenocytes at the highest tested concentration, even after four days of treatment. Moreover, mechanistic studies showed that the treatment with this compound promoted oxidative stress in the cells of the parasites and a type of apoptosis-like cell death [124].

**Scheme 22:**
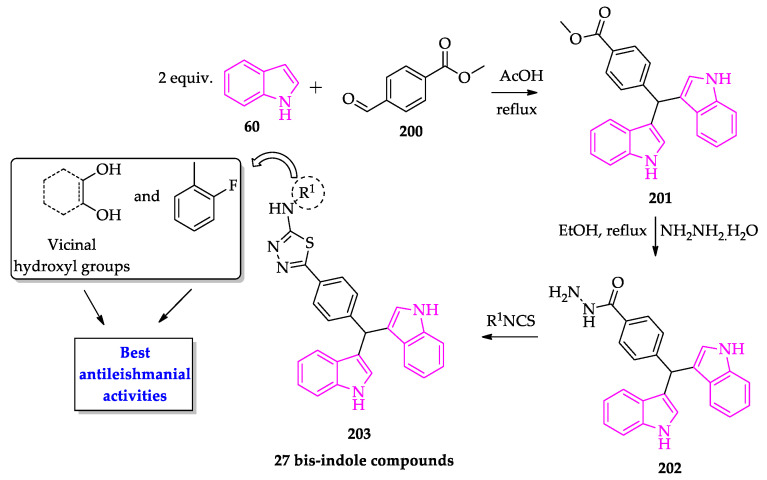
Synthesis of bis-indole derivatives **203** with antileishmanial activity.

Inspired by the biological potential of natural bis-indole compounds, Taha et al. synthetized 27 synthetic bis-indole analogs and investigated their antileishmanial activity [125]. The compounds were obtained *via* a simple three-step procedure, starting with the condensation of two equivalents of indole with methyl-4-formylbenzoate (**200**) (Scheme 22) [125]. In sequence, the intermediate was treated with hydrazine hydrate to generate the acyl hydrazide that was converted to the final products **203** by the reaction with different isothiocyanates [125]. In comparison to the standard drug (pentamidine), most derivatives potently inhibited the in vitro growth of the parasite. The SAR study was able to identify structural features that most impacted the activity. In particular, the presence of vicinal dihydroxy systems is the most relevant feature [125]. Docking studies with the most active compound indicated that compounds of this class tend to interact with key residues on pteridine reductase, which could explain the inhibitory activity [125].

**Scheme 23:**
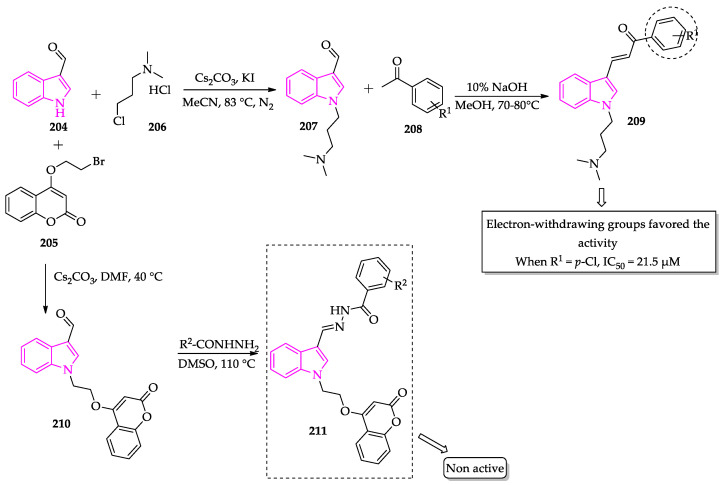
Synthesis of *N*-substituted indole hybrids.

Tiwari et al. studied the pharmacological properties of a group of *N*-substituted indole derivatives [126]. Using different synthetic approaches, the authors accomplished the synthesis of chalcone-based and hydrazine–hydrazide derivatives (Scheme 23). It was observed that chalcone-based compounds exhibited the best activities regarding antileishmanial activity, particularly compounds with electron-withdrawing substituents (e.g., *p*-chloro, IC_50_ = 21.5 µM) [126].

**Scheme 24:**
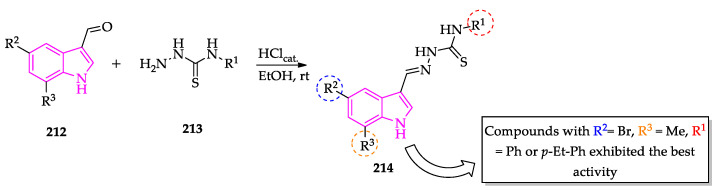
Procedure to obtain indol-3-yl-thiosemicarbazone derivatives with antileishmanial activity.

Silva et al. derivatized the indole nucleus by the introduction of thiosemicarbazone, another biologically privileged group with antileishmanial properties. For this, they reacted substituted indole-carboxaldehyde with previously synthetized thiosemicarbazides (Scheme 24) [127]. The authors investigated the activity against two species of *Leishmania* parasites, *L. infantum* and *L. amazonensis* [127]. In general, the compounds were shown to be more active against *L. infantum* promastigotes, with IC_50_ ranging from 4.36 µM to 23.25 µM. In general, the synthetized compounds exhibited low cytotoxicity against the murine macrophage J774 cell line (CC_50_ ranging from 53.23 µM to 357.97 µM). From this series of compounds, it was possible to identify that the substitution pattern in the phenyl ring of the thiosemicarbazone moiety was relevant for the antileishmanial activity [127].

**Scheme 25:**
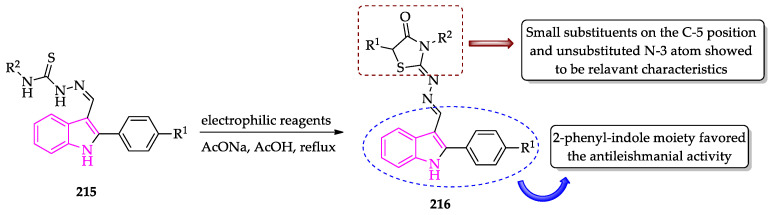
Synthesis of thiazolidinonephenyl–indole hybrids with antileishmanial activity.

Interestingly, Schadich et al. demonstrated that the cyclization of indole-based thiosemicarbazone appears to be another strategy for generating compounds with promising antileishmanial activity (Scheme 25). It should be noted that these compounds had already demonstrated antitrypanosomal activity [91]. Among the most active compounds bearing a thiazolidinone unit **216** presented the clearest structure–activity relationships in assays against *L. major* FV1 promastigotes [128]. Particularly, it was identified that activity was strongly dependent on the presence of small substituents on the C-5 position of the thiazolidinone core or the absence of substituents on the N-3 position [128].

## 3. Conclusions

Parasitic diseases, such as malaria, trypanosomiasis and leishmaniasis, are still a cause of great health, social and economic burden in endemic countries and cause a considerable number of preventable deaths around the world, especially in tropical and subtropical countries. In addition to problems with toxicity and low efficacy, the emergence of drug resistance has further complicated the already limited clinical management of these diseases. From a medicinal chemistry perspective, it is of utmost interest to identify new chemotypes that exhibit different mechanisms of action than the drugs already used in the clinic. In this review, we present several pieces of evidence that support the high pharmacological potential of indole-based compounds for the development of drugs to treat these diseases. A concrete example is the spiroindolone cipargamin (**91**) (KAE609), an inhibitor of the *Plasmodium falciparum* P-type ATPase 4, which has successfully completed phase II studies for malaria treatment. Despite having good antiparasitic activity, in most cases, the mechanism of action of indole-based compounds is not properly investigated. Therefore, we expect that this review will encourage more medicinal chemists to continue investing their creative strength in the discovery of new indole-based antiparasitic agents with novel mechanisms of action.

## Data Availability

Not applicable.
